# Erectile Dysfunction Drugs as Potential Therapy for Cognitive Decline: Preclinical and Translational Evidence

**DOI:** 10.3390/cells14191505

**Published:** 2025-09-26

**Authors:** Roberta Ricciarelli

**Affiliations:** 1 Section of General Pathology, Department of Experimental Medicine, School of Medical and Pharmacological Sciences, University of Genoa, 16132 Genoa, Italy; ricciarelli@medicina.unige.it; 2 IRCCS Ospedale Policlinico San Martino, 16132 Genoa, Italy

**Keywords:** phosphodiesterases, vardenafil, sildenafil, NO/cGMP, Alzheimer’s disease

## Abstract

Erectile dysfunction (ED) and cognitive decline share overlapping vascular, metabolic, and neurodegenerative mechanisms, particularly in aging populations. Phosphodiesterase type 5 inhibitors (PDE5-Is), such as sildenafil and vardenafil, are widely used to treat ED by elevating cyclic guanosine monophosphate (cGMP) levels and enhancing vascular function. Emerging evidence suggests that PDE5-Is may also benefit cognitive function by promoting neurovascular coupling, synaptic plasticity, and neuroprotection. This review synthesizes clinical, preclinical, and mechanistic studies on PDE5-Is in the context of learning, memory, and Alzheimer’s disease, highlighting their potential as therapeutic agents for cognitive impairment.

## 1. Introduction: Linking Erectile Dysfunction, Cognitive Decline, and NO-cGMP Signaling

Epidemiological studies have consistently shown that the prevalence of erectile dysfunction (ED) increases with age and is associated with cardiovascular, metabolic, and neurological conditions [1,2]. Similarly, cognitive decline arises from both neurodegenerative and vascular mechanisms, with metabolic factors also contributing. Interestingly, the nitric oxide (NO)-cyclic guanosine monophosphate (cGMP) signaling pathway, which mediates penile erection, also acts as a neurotransmitter system in the central nervous system (CNS), where it plays key roles in synaptic plasticity and memory formation [3]. This physiological overlap has prompted growing interest in repurposing phosphodiesterase type 5 inhibitors (PDE5-Is) as potential cognitive enhancers.

This review highlights key mechanistic and clinical aspects of this emerging connection, examining preclinical and clinical evidence supporting the cognitive effects of PDE5-Is and their potential therapeutic role in neurodegenerative and age-related cognitive disorders.

Literature searches were conducted in PubMed using combinations of the following keywords: PDE5 inhibitors, sildenafil, tadalafil, cognition, Alzheimer’s disease, vascular dementia, NO-cGMP signaling, and neuroprotection. Searches included all articles published up to July 2025, with no language restrictions. Reference lists of relevant studies and reviews were also screened. Inclusion criteria were studies (preclinical, clinical, mechanistic) investigating PDE5-Is in the context of cognition, Alzheimer’s disease (AD), or neurodegeneration; exclusion criteria included studies unrelated to cognitive or neurological outcomes.

## 2. Mechanisms of NO-cGMP Signaling in Neurons and Vasculature: Implications for PDE5 Inhibition

cGMP is synthesized by two classes of guanylyl cyclase: particulate guanylyl cyclases (pGCs) and soluble guanylyl cyclase (sGC), which differ in both localization and activation mechanisms [4]. pGCs are membrane-bound enzymes activated by specific ligands (e.g., natriuretic peptides), whereas sGC is a cytosolic enzyme activated by NO and widely expressed throughout the body, with particularly high levels in the CNS.

In neurons, cGMP activates protein kinase G (PKG), which phosphorylates various downstream targets, including CREB, a critical transcription factor involved in long-term potentiation (LTP). Additionally, cGMP directly modulates neuronal excitability and synaptic transmission through its action on ion channels, particularly cyclic nucleotide-gated (CNG) channels and hyperpolarization-activated cyclic nucleotide-modulated (HCN) channels [5,6]. These channels contribute to the regulation of sensory input and neuronal rhythmicity, respectively. Notably, in hippocampal tissue and neuronal cultures, cGMP has also been described as a modulator of amyloid-β (Aβ) production, suggesting a potential role in regulating AD-related pathology [4,7,8].

The signaling activity of cGMP is terminated by its hydrolysis into 5′ GMP, a reaction catalyzed by phosphodiesterases (PDEs). Among the 11 PDE families, PDE5, PDE6, and PDE9 are selective for cGMP. Unlike PDE6, which is primarily retinal, or PDE9, which lacks regulatory domains, PDE5 is integrated into feedback-regulated cGMP signaling pathways. Particularly, PKG-mediated phosphorylation enhances PDE5′s affinity for cGMP, forming a feed-forward regulatory loop. Moreover, PDE5 is believed to function within specialized signaling microdomains (signalosomes), allowing for precise spatial control of cGMP activity [9,10].

PDE5 is highly expressed in the smooth muscle cells of the peripheral arteries and veins, including coronary and pulmonary vessels [11]. It is also abundant in the vascular smooth muscle cells of the corpus cavernosum of the penis. Beyond these sites, PDE5 is found in myometrial cells, endothelial cells and peripheral blood mononuclear cells, and is expressed in several other tissues such as skeletal muscles, cardiomyocytes, platelets, lung, spinal cord, cerebellum, retina, pancreas, prostate, urethra and bladder [12,13].

The clinical use of PDE5-Is is primarily based on their action in vascular tissues, where inhibition of PDE5 leads to increased levels of cGMP, promoting vasodilatation. In the context of cerebral vasculature, PDE5-Is are therefore expected to enhance brain perfusion and support metabolic health, providing a rationale for investigating their impact on cerebrovascular regulation and neurovascular coupling, particularly in conditions where cerebral blood flow is compromised [14,15]. However, other PDE families also contribute to cGMP signaling in the brain. For instance, PDE9, despite lacking GAF domains, is highly expressed in the hippocampus and cortex and plays a critical role in regulating basal cGMP tone, particularly in glutamatergic neurons [16,17]. PDE1, PDE2, PDE3, and PDE4 further modulate cyclic nucleotide signaling and synaptic plasticity through cAMP-cGMP crosstalk [18]. Inhibitors of these PDEs have shown cognitive-enhancing effects in preclinical studies, and combination approaches with PDE5-Is have additive or synergistic effects on cognition [19,20]. This highlights that PDE5 inhibition represents one of multiple pharmacological strategies to elevate cGMP signaling in the CNS and suggests that future therapies may benefit from multi-target PDE modulation.

Although PDE5 has been the primary therapeutic target in the context of ED and vascular regulation, a direct neuronal effect should also be considered, as PDE5 has been reported in neurons, particularly in brain regions involved in cognition, such as the cortex and hippocampus [21,22]. Evidence from human studies, however, is mixed: several reports found very low or undetectable PDE5 mRNA in brain tissue [22,23,24,25], though discrepancies may reflect methodological limitations. For example, one negative study employed a rodent probe to detect human transcript [25]. Subsequent analyses with species-specific primers confirmed PDE5 mRNA in the human brain, and protein expression was validated by Western blot and ELISA [21]. Immunohistochemistry further localized PDE5 to cortical and hippocampal neurons [21].

Notably, in rodents, cGMP-PDE activity in the hippocampus and cortex appears to increase with aging [26], although age-dependent changes in PDE5 mRNA expression were not observed [27].

**Figure 1:**
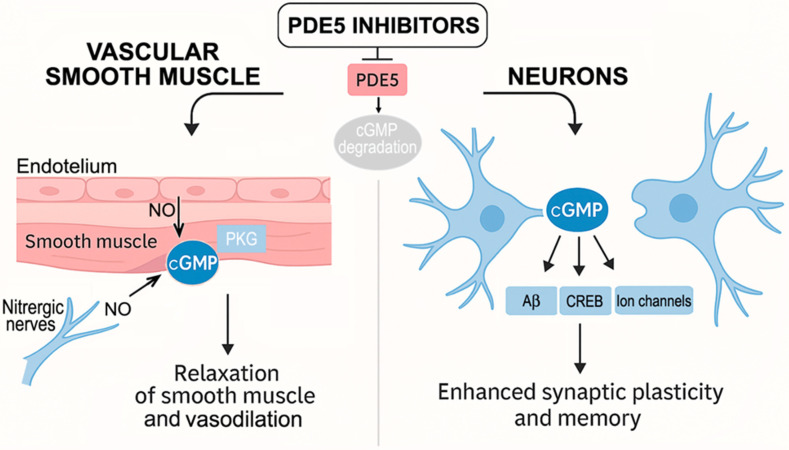
Dual role of PDE5-Is in enhancing neuronal and vascular cGMP signaling. NO released by neurons and endothelial cells activates sGC, which catalyzes the conversion of GTP to cGMP. cGMP then activates PKG. In vascular smooth muscle, PKG reduces intracellular calcium levels, leading to smooth muscle relaxation and vasodilation. In central neurons, PKG phosphorylates various targets, including the transcription factor CREB. Additionally, cGMP can directly modulate ion channels and stimulate the release of Aβ peptides [5,6,7,8]. PDE5 regulates cGMP levels by hydrolyzing it into the inactive 5′ GMP. Therefore, PDE5-Is enhance cGMP signaling in both the vascular and central nervous systems. NO, the smallest known signaling molecule, is synthesized by three isoforms of nitric oxide synthase (NOS): neuronal NOS (nNOS), inducible NOS (iNOS), and endothelial NOS (eNOS).

Together, these findings suggest that, in addition to their cerebrovascular effects, PDE5-Is may also enhance NO-mediated synaptic plasticity, offering potential cognitive benefits (Figure 1).

nNOS is constitutively expressed in specific neurons in the brain and exists in both particulate and soluble forms, enabling distinct subcellular distribution that may underpin diverse functional roles. Beyond the CNS, nNOS is also present in the spinal cord, epithelial cells, sympathetic ganglia, adrenal glands, peripheral nerves, kidney, pancreas, and skeletal muscle [28,29,30].

In the periphery, many smooth muscle tissues are innervated by nitrergic (nNOS-containing) nerves, which release NO to activate sGC and induce smooth muscle relaxation via the cGMP pathway. Therefore, although eNOS has traditionally been viewed as the primary modulator of peripheral vascular tone, it is now evident that nNOS plays a parallel and significant role, independent of its CNS function [31]. Moreover, vascular smooth muscle cells themselves express low levels of nNOS, which contribute to vasodilation, particularly in conditions where eNOS is dysfunctional [30].

Penile erection is a well-characterized example of NO-cGMP signaling in peripheral tissues: relaxation of smooth muscle in the corpus cavernosum is mediated by nitrergic nerves and cGMP accumulation [32]. PDE5, the major cGMP-degrading enzyme in this tissue, serves as the target for PDE5-Is, which prolong cGMP activity and facilitate erectile function.

## 3. PDE5-Is

**Figure 2:**
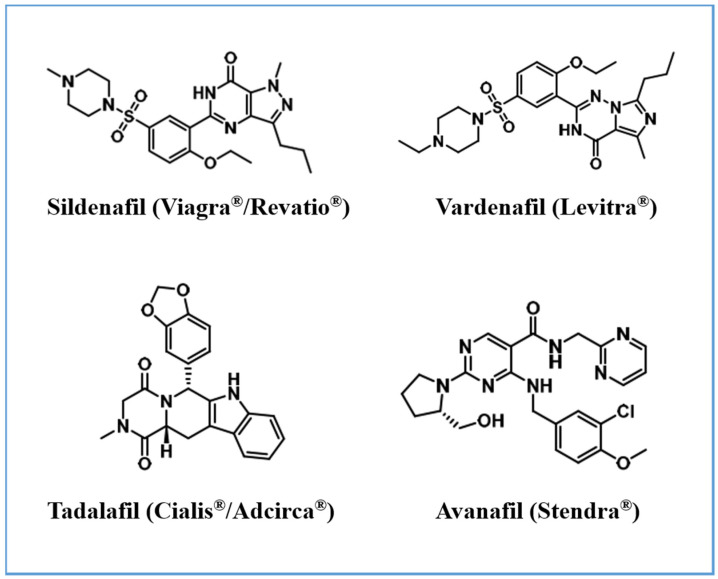
Chemical structure of FDA-approved PDE5 inhibitors.

Several PDE5-Is have been approved by the U.S. Food and Drug Administration (FDA) for clinical use. These include: (i) Sildenafil, first approved in 1998 for erectile dysfunction (ED) under the trade name Viagra^®^, and later in 2005 for pulmonary arterial hypertension (PAH) as Revatio^®^; (ii) Vardenafil, approved in 2003 for ED as Levitra^®^; (iii) Tadalafil, which gained approval in 2003 for ED as Cialis^®^, in 2009 for PAH as Adcirca^®^, and in 2011 for the treatment of lower urinary tract symptoms linked to benign prostatic hyperplasia; (iv) Avanafil, the most recent addition, approved in 2012 for ED as Stendra^®^ [33] (Figure 2).

Among them, vardenafil exhibits the highest selectivity for PDE5, with reported IC50 values between 0.1 and 0.4 nM, followed by tadalafil (IC50 ~2 nM), and then sildenafil and avanafil, with IC50 values of approximately 4 and 4.3–5.2 nM, respectively [34].

Although PDE5-Is are administered peripherally, preclinical evidence indicates that sildenafil and vardenafil can cross the blood–brain barrier and reach concentrations sufficient to inhibit PDE5 in the CNS. PET and ex vivo studies in rodents demonstrated CNS penetration by sildenafil [35,36], while pharmacokinetic analyses in non-human primates showed that oral administration achieves cerebrospinal fluid (CSF) levels capable of elevating cGMP [35]. Similarly, vardenafil treatment in rats confirmed brain accumulation, supporting CNS target engagement [37]. However, pharmacokinetic data in humans remain limited, highlighting the need for further research to clarify CNS exposure and optimize dosing strategies.

Several novel PDE5-I have been developed in recent years and are being explored for a wide range of potential indications, including cardiovascular disease, cancer, chronic kidney disease, cystic fibrosis, and diabetes [38]. Promising preclinical findings also suggest potential cognitive benefits, as discussed in the following section. In addition to these research efforts, some newer-generation PDE5-Is, such as lodenafil, udenafil, and mirodenafil, are already marketed in countries like Brazil and South Korea for the treatment of erectile dysfunction, although they have not yet received FDA approval.

## 4. Cognitive Effects of PDE5-Is

### 4.1. Preclinical Evidence

A growing body of preclinical research indicates that the inhibition of PDE5 has a positive impact on cognitive function across various experimental models, both under healthy conditions and in models of aging or neurodegeneration. In particular, studies on hippocampal slices have shown that both sildenafil and vardenafil can convert transient early-phase LTP (E-LTP) into the more stable late-phase LTP (L-LTP), a cellular correlate of long-term memory consolidation [8,39]. Furthermore, behavioral studies have demonstrated memory-enhancing effects of PDE5-Is in healthy rodents and non-human primates following both acute and chronic administration [40,41,42].

In disease models, the benefits of PDE5 inhibition appear to extend beyond synaptic enhancement. For example, chronic treatment with sildenafil in Tg2576 transgenic mice, a widely used model of AD, fully reversed cognitive deficits without affecting Aβ burden. Mechanistic investigations revealed that this effect was mediated through inhibition of tau hyperphosphorylation, likely via suppression of glycogen synthase kinase 3β (GSK3β) and cyclin-dependent kinase 5 (CDK5) activity [43]. In a different AD model (APP/PS1 transgenic mice), long-term sildenafil administration improved synaptic function and cognitive performance by boosting cGMP/PKG/CREB signaling, suppressing neuroinflammation, and lowering hippocampal Aβ levels [36,44]. However, the role of Aβ in mediating the effects of PDE5-Is remains controversial, as evidence suggests that Aβ itself may be necessary for the pro-cognitive effects of cGMP signaling [8,45]. Notably, studies reporting beneficial effects of PDE5-Is on Aβ reduction have primarily been conducted in transgenic models of Aβ overproduction [36,44,46]. This raises the possibility that cGMP signaling may act differently under physiological versus pathological Aβ levels. Both Aβ and cGMP display hormetic, dose-dependent effects [47,48], and NO donors also modulate APP processing bidirectionally [49]. High Aβ concentrations reduce cGMP in multiple models [50,51,52], and in mild AD patients, lower CSF cGMP correlates with cognitive decline and reduced Aβ42 [53]. Together, these findings support a cGMP–Aβ feedback loop, where cGMP promotes Aβ production, which in turn suppresses cGMP, potentially explaining the Aβ-lowering effects of chronic PDE5-I treatment in AD models.

Among the various PDE5-Is, tadalafil has the longest half-life, with efficacy in ED lasting up to 36 h [34], potentially offering therapeutic advantages beyond ED when compared to shorter-acting agents. Tested chronically, tadalafil treatment improved spatial memory and reduced tau pathology in the J20 mouse model of AD [54], as well as decreased hippocampal oxidative stress in aged mice [55].

PDE5-Is have also been implicated in providing neuroprotection under conditions of stress or injury. For instance, sildenafil has been shown to enhance performance in novel object recognition tasks and increase brain-derived neurotrophic factor (BDNF) levels in models with experimentally induced cognitive impairments [56,57]. Moreover, under noise-induced stress, sildenafil attenuated hippocampal cognitive decline and improved antioxidant defenses [58].

Several newer-generation PDE5-Is have shown promising neuroprotective and cognitive effects in preclinical models. For instance, the flavonoid icariside II significantly attenuated cognitive deficits induced by Aβ25–35 injection in rats [59]. In a mouse model of tauopathy, the pyrazoline derivative RF26 improved spatial memory and reduced phosphorylated tau accumulation, gliosis, and pro-inflammatory cytokine levels [60]. Similarly, mirodenafil, a pyrrolopyrimidinone-based compound, enhanced cognitive performance in both APP-C105 [61] and ApoE4 knock-in [62] mouse models of AD.

Novel quinoline-based PDE5-Is have also demonstrated efficacy in AD models: compound 4b restored synaptic plasticity in electrophysiological studies [63], while compound 6c reversed memory deficits in behavioral tasks [64].

In addition, dual-acting compounds targeting PDE5 and additional pathways, such as histone deacetylase [65,66] or Rho-associated protein kinase (ROCK) [67], have further improved cognitive outcomes in animal models.

### 4.2. Clinical Evidence

Recent reviews have summarized findings from clinical trials on PDE5-Is for cognitive enhancement and AD [68,69,70]. However, none of these compounds has yet received regulatory approval for such indications.

In cognitively healthy individuals, preliminary evidence suggests that sildenafil may improve reaction time and attention, as reflected in auditory event-related potentials, although consistent benefits on memory have not been demonstrated [71,72]. In patients with AD, a single dose of sildenafil has been shown to enhance cerebral hemodynamics [73] and reduce spontaneous neural hyperactivity [74], a parameter aberrantly elevated in the hippocampus and parahippocampal regions, while no comparable effects have been reported in individuals with schizophrenia [75].

Also, findings for other PDE5-Is have been mixed. Low single doses of vardenafil (10 or 20 mg) did not alter auditory event-related potentials or memory performance in healthy young adults [76,77]. By contrast, longer treatment regimens have shown promise, particularly with udenafil [78,79] and tadalafil [80,81].

Table 1 summarizes the relatively few clinical trials investigating PDE5-I and cognitive outcomes. These studies are limited by small cohort sizes, short intervention periods, and possibly suboptimal participant selection; however, they leave open the possibility that larger, well-powered trials may demonstrate disease-modifying benefits in AD or vascular cognitive impairments.

More supportive evidence comes from recent epidemiological studies. An analysis of data from 7.23 million U.S. insurance policyholders found that sildenafil use was associated with a 69% reduced risk of developing AD [82]. This result was corroborated by a subsequent population-based cohort study reporting that men with erectile dysfunction who were prescribed PDE5-Is had a reduced incidence of AD, with the strongest effect observed in those receiving more frequent prescriptions [83].

However, not all findings align with this optimistic picture. A study by Desai et al. in patients with PAH found no significant association between PDE5-I use and the risk of AD-related dementia (ADRD) [84]. In this analysis, patients treated with sildenafil or tadalafil were compared to those receiving an endothelin receptor antagonist (ERA), and no significant difference in ADRD incidence was observed. While this study had notable strengths, including careful matching of treatment and comparator groups, several limitations have been identified [69]. PAH is a rare, severe disease that typically affects individuals under the age of 60 and is associated with poor prognosis, limiting the generalizability of the findings. The relatively small, highly specific Medicare claims population may have been underpowered to detect differences in dementia risk. Moreover, the short follow-up period (approximately 6 months) was likely insufficient to capture meaningful neurodegenerative changes, and PDE5-I doses may not have reached therapeutically relevant CNS concentrations. Notably, ERAs, the class of comparator drugs, have recently been linked to a reduced risk of AD [85], raising the possibility that the study compared two potentially protective treatments, thereby diminishing detectable differences.

## 5. Adverse Effects of PDE5-Is

The primary contraindication for PDE5-Is is their concurrent use with organic nitrates, which are prescribed for conditions like angina and heart failure. This combination can lead to severe hypotension [86], though a large observational study found no significant difference in cardiovascular outcomes between patients using both medications and those taking nitrates alone [87]. Additionally, tadalafil combined with antihypertensive medications has not been linked to increased cardiovascular risk [88]. Clinical guidelines, however, recommend against the use of PDE5-Is in cases of advanced congestive heart failure, unstable or treatment-resistant angina pectoris, recent myocardial infarction, high-risk arrhythmias, obstructive hypertrophic cardiomyopathy, and severe valve diseases, particularly aortic stenosis [89].

PDE5-Is also exhibit weak inhibition of PDE6 in retinal photoreceptors, which can occasionally lead to transient visual disturbances [90]. Moreover, as PDE5 is expressed in choroidal and retinal vessels, its inhibition may enhance ocular blood flow, potentially contributing to rare complications such as non-arteritic anterior ischemic optic neuropathy (NAION), chorioretinopathy, glaucoma, and optic atrophy [90]. A placebo-controlled trial involving healthy men demonstrated reversible changes in retinal function following a single 100 mg sildenafil dose [91]. Importantly, a subsequent meta-analysis reported no significant association with NAION, suggesting an overall acceptable ocular safety profile for PDE5-Is [92].

Other minor adverse events associated with these medications include mild vasodilatory reactions such as headache, flushing, dyspepsia, and nasal congestion or rhinitis.

## 6. Conclusions

Preclinical evidence from in vitro studies and animal models consistently supports the neuroprotective and cognitive-enhancing effects of PDE5-Is. Large-scale clinical datasets also suggest potential benefit; however, findings remain inconsistent, likely due to methodological limitations such as small sample sizes, insufficient treatment durations, and heterogeneous patient populations. To conclusively determine the therapeutic potential of PDE5-Is in AD and other cognitive impairments, well-powered, randomized clinical trials are urgently needed. These trials must be rigorously designed with appropriate stratification by disease subtype and stage, clearly defined endpoints, and inclusion of both female and male participants. Sex differences may be a critical determinant of therapeutic response. Most clinical studies on PDE5-Is have focused on male populations, primarily because of their original indication for erectile dysfunction, leaving the effects in females largely unexplored. Yet, cognitive decline affects both sexes, and preclinical studies suggest that sex hormones can modulate PDE5 expression and downstream cGMP signaling [93,94], potentially influencing both efficacy and optimal dosing. Inclusion of female participants and stratified analyses by sex are therefore essential to identify potential differential responses, tailor treatment strategies, and avoid sex-based bias in interpreting clinical outcomes.

Critical questions remain. What is the optimal therapeutic window during which PDE5-Is exert maximal benefit? Which biomarker panels can best guide patient selection and trial design? Moreover, for approved PDE5-Is such as sildenafil and vardenafil, definitive data on CNS penetration in humans are lacking. Resolving this issue is crucial to determining whether repurposing these compounds is viable or whether development should shift toward novel molecules optimized for CNS applications, particularly those with improved brain permeability, longer half-lives, and greater selectivity for PDE5 over related isoforms (e.g., PDE6, PDE11).

A central unresolved question concerns the primary mechanism of PDE5-Is in the brain (Figure 1): Are their effects mediated through vascular PDE5, mirroring their action in ED and PAH, or through direct neuronal PDE5 modulation? Clarifying this distinction will help define the ideal patient population, whether PDE5-Is are most effective in individuals with mixed AD and cerebrovascular pathology, purely neurodegenerative disease, or vascular dementia without amyloid involvement.

As our understanding of the molecular drivers of cognitive decline deepens, PDE5-Is may yet emerge as a valuable, if unexpected, tool in the treatment of dementia. Given their mechanistic plausibility, preclinical efficacy, and established clinical safety profiles, these compounds merit continued and rigorous investigation, particularly in the context of AD and vascular cognitive impairment.

## Figures and Tables

**Table 1:** PDE5-Is in clinical trials assessing cognition.

COMPOUND	POPULATION	DOSE	MAIN OUTCOMES	REFS
**Sildenafil**	Older adults with AD N = 10 (5 females)	50 mg Single dose	Possible positive effects	[74]
	Older adults with AD N = 12 (7 females)	50 mg Single dose	Possible positive effects	[73]
	Adults with schizophrenia N = 18 (10 females)	50/100 mg Single dose	No effect	[75]
	Healthy males N = 6	100 mg Single dose	No effect	[72]
	Healthy males N = 10	100 mg Single dose	No effect	[71]
**Vardenafi**	Healthy young adults N = 18 (13 females)	10/20 mg Single dose	No effect	[76,77]
**Udenafil**	ED patients N = 27	100 mg/day for 4 months	Positive effects	[78]
	ED patients N = 49	50 mg/day for 2 months	Positive effects	[79]
**Tadalafil**	Adult males with ED and MCI N = 25	5 mg/day for 8 weeks	Positive effects	[80]
	Adult males with BPH/LUTS-ED (N = 9), healthy controls (N = 12)	5 mg/day for 6 months	Positive effects	[81]

AD: Alzheimer’s disease, ED: erectile dysfunction, MCI: mild cognitive impairment, BPH: benign prostatic hyperplasia, LUTS: low urinary tract symptoms.

## Data Availability

No new data were created or analyzed in this study.

## References

[1] Lewis R.W., Fugl-Meyer K.S., Corona G., Hayes R.D., Laumann E.O., Moreira E.D., Rellini A.H., Segraves T. (2010). Original Articles: Definitions/Epidemiology/Risk Factors for Sexual Dysfunction. J. Sex. Med..

[2] Jannini E.A., Sternbach N., Limoncin E., Ciocca G., Gravina G.L., Tripodi F., Petruccelli I., Keijzer S., Isherwood G., Wiedemann B. (2014). Health-Related Characteristics and Unmet Needs of Men with Erectile Dysfunction: A Survey in Five European Countries. J. Sex. Med..

[3] Feil R., Kleppisch T. (2008). NO/cGMP-dependent modulation of synaptic transmission. Handb. Exp. Pharmacol..

[4] Fedele E., Ricciarelli R. (2021). Memory Enhancers for Alzheimer’s Dementia: Focus on cGMP. Pharmaceuticals.

[5] Podda M.V., Grassi C. (2014). New perspectives in cyclic nucleotide-mediated functions in the CNS: The emerging role of cyclic nucleotide-gated (CNG) channels. Pflügers Arch. Eur. J. Physiol..

[6] Herrmann S., Schnorr S., Ludwig A. (2015). HCN Channels—Modulators of Cardiac and Neuronal Excitability. Int. J. Mol. Sci..

[7] Calcagno E., Caudano F., Passalacqua M., Pronzato M.A., Fedele E., Ricciarelli R. (2017). Investigating the amyloid-beta enhancing effect of cGMP in neuro2a cells. Mech. Ageing Dev..

[8] Palmeri A., Ricciarelli R., Gulisano W., Rivera D., Rebosio C., Calcagno E., Tropea M.R., Conti S., Das U., Roy S. (2017). Amyloid-β Peptide Is Needed for cGMP-Induced Long-Term Potentiation and Memory. J. Neurosci..

[9] Maurice D.H., Wilson L.S., Rampersad S.N., Hubert F., Truong T., Kaczmarek M., Brzezinska P., Freitag S.I., Umana M.B., Wudwud A. (2014). Cyclic nucleotide phosphodiesterases (PDEs): Coincidence detectors acting to spatially and temporally integrate cyclic nucleotide and non-cyclic nucleotide signals. Biochem. Soc. Trans..

[10] Brescia M., Zaccolo M. (2016). Modulation of Compartmentalised Cyclic Nucleotide Signalling via Local Inhibition of Phosphodiesterase Activity. Int. J. Mol. Sci..

[11] Lin C.S., Lin G., Xin Z.C., Lue T. (2006). Expression, Distribution and Regulation of Phosphodiesterase 5. Curr. Pharm. Des..

[12] Francis S.H., Blount M.A., Corbin J.D. (2011). Mammalian Cyclic Nucleotide Phosphodiesterases: Molecular Mechanisms and Physiological Functions. Physiol. Rev..

[13] Ückert S., Waldkirch E.S., Merseburger A.S., Kuczyk M.A., Oelke M., Hedlund P. (2013). Phosphodiesterase type 5 (PDE5) is co-localized with key proteins of the nitric oxide/cyclic GMP signaling in the human prostate. World J. Urol..

[14] Pauls M.M., Moynihan B., Barrick T.R., Kruuse C., Madigan J.B., Hainsworth A.H., Isaacs J.D. (2018). The effect of phosphodiesterase-5 inhibitors on cerebral blood flow in humans: A systematic review. J. Cereb. Blood Flow Metab..

[15] AlRuwaili R., Al-Kuraishy H.M., Alruwaili M., Khalifa A.K., Alexiou A., Papadakis M., Saad H.M., Batiha G.E.-S. (2024). The potential therapeutic effect of phosphodiesterase 5 inhibitors in the acute ischemic stroke (AIS). Mol. Cell. Biochem..

[16] Harms J.F., Menniti F.S., Schmidt C.J. (2019). Phosphodiesterase 9A in Brain Regulates cGMP Signaling Independent of Nitric-Oxide. Front. Neurosci..

[17] Ishihara Y., Ando M., Goto Y., Kotani S., Watanabe N., Nakatani Y., Ishii S., Miyamoto N., Mano Y., Ishikawa Y. (2025). A novel selective phosphodiesterase 9 inhibitor, irsenontrine (E2027), enhances GluA1 phosphorylation in neurons and improves learning and memory via cyclic GMP elevation. Neuropharmacology.

[18] Nabavi S.M., Talarek S., Listos J., Devi K.P., de Oliveira M.R., Tewari D., Argüelles S., Mehrzadi S., Hosseinzadeh A., D’Onofrio G. (2019). Phosphodiesterase inhibitors say NO to Alzheimer’s disease. Food Chem. Toxicol..

[19] Gulisano W., Tropea M.R., Arancio O., Palmeri A., Puzzo D. (2018). Sub-efficacious doses of phosphodiesterase 4 and 5 inhibitors improve memory in a mouse model of Alzheimer’s disease. Neuropharmacology.

[20] Park M.K., Yang H.W., Woo S.Y., Kim D.Y., Son D.-S., Choi B.Y., Suh S.W. (2025). Modulation of Second Messenger Signaling in the Brain Through PDE4 and PDE5 Inhibition: Therapeutic Implications for Neurological Disorders. Cells.

[21] Teich A.F., Sakurai M., Patel M., Holman C., Saeed F., Fiorito J., Arancio O. (2016). PDE5 Exists in Human Neurons and is a Viable Therapeutic Target for Neurologic Disease. J. Alzheimer’s Dis..

[22] Lakics V., Karran E.H., Boess F.G. (2010). Quantitative comparison of phosphodiesterase mRNA distribution in human brain and peripheral tissues. Neuropharmacology.

[23] Loughney K., Hill T.R., Florio V.A., Uher L., Rosman G.J., Wolda S.L., Jones B.A., Howard M.L., McAllister-Lucas L.M., Sonnenburg W.K. (1998). Isolation and characterization of cDNAs encoding PDE5A, a human cGMP-binding, cGMP-specific 3′,5′-cyclic nucleotide phosphodiesterase. Gene.

[24] Yanaka N., Kotera J., Ohtsuka A., Akatsuka H., Imai Y., Michibata H., Fujishige K., Kawai E., Takebayashi S., Okumura K. (1998). Expression, structure and chromosomal localization of the human cGMP-binding cGMP-specific phosphodiesterase PDE5A gene. Eur. J. Biochem..

[25] Reyes-Irisarri E., Markerink-Van Ittersum M., Mengod G., de Vente J. (2007). Expression of the cGMP-specific phosphodiesterases 2 and 9 in normal and Alzheimer’s disease human brains. Eur. J. Neurosci..

[26] Kelly M.P. (2018). Cyclic nucleotide signaling changes associated with normal aging and age-related diseases of the brain. Cell. Signal..

[27] Kelly M.P., Adamowicz W., Bove S., Hartman A.J., Mariga A., Pathak G., Reinhart V., Romegialli A., Kleiman R.J. (2014). Select 3′,5′-cyclic nucleotide phosphodiesterases exhibit altered expression in the aged rodent brain. Cell. Signal..

[28] Zhou L., Zhu D.Y. (2009). Neuronal nitric oxide synthase: Structure, subcellular localization, regulation, and clinical implications. Nitric Oxide.

[29] Nakane M., Schmidt H.H.H.W., Pollock J.S., Förstermann U., Murad F. (1993). Cloned human brain nitric oxide synthase is highly expressed in skeletal muscle. FEBS Lett..

[30] Schwarz P.M., Kleinert H., Förstermann U. (1999). Potential Functional Significance of Brain-Type and Muscle-Type Nitric Oxide Synthase I Expressed in Adventitia and Media of Rat Aorta. Arter. Thromb. Vasc. Biol..

[31] Melikian N., Seddon M.D., Casadei B., Chowienczyk P.J., Shah A.M. (2009). Neuronal Nitric Oxide Synthase and Human Vascular Regulation. Trends Cardiovasc. Med..

[32] Dean R.C., Lue T.F. (2005). Physiology of Penile Erection and Pathophysiology of Erectile Dysfunction. Urol. Clin. N. Am..

[33] Bruzziches R., Francomano D., Gareri P., Lenzi A., Aversa A. (2013). An update on pharmacological treatment of erectile dysfunction with phosphodiesterase type 5 inhibitors. Expert Opin. Pharmacother..

[34] Gupta M., Kovar A., Meibohm B. (2005). The Clinical Pharmacokinetics of Phosphodiesterase-5 Inhibitors for Erectile Dysfunction. J. Clin. Pharmacol..

[35] Gómez-Vallejo V., Ugarte A., García-Barroso C., Cuadrado-Tejedor M., Szczupak B., Dopeso-Reyes I.G., Lanciego J.L., García-Osta A., Llop J., Oyarzabal J. (2016). Pharmacokinetic investigation of sildenafil using positron emission tomography and determination of its effect on cerebrospinal fluid cGMP levels. J. Neurochem..

[36] Puzzo D., Staniszewski A., Deng S.X., Privitera L., Leznik E., Liu S., Zhang H., Feng Y., Palmeri A., Landry D.W. (2009). Phosphodiesterase 5 Inhibition Improves Synaptic Function, Memory, and Amyloid-beta Load in an Alzheimer’s Disease Mouse Model. J. Neurosci..

[37] Reneerkens O.A., Rutten K., Akkerman S., Blokland A., Shaffer C.L., Menniti F.S., Steinbusch H.W., Prickaerts J. (2012). Phosphodiesterase type 5 (PDE5) inhibition improves object recognition memory: Indications for central and peripheral mechanisms. Neurobiol. Learn. Mem..

[38] ElHady A.K., El-Gamil D.S., Abdel-Halim M., Abadi A.H. (2023). Advancements in Phosphodiesterase 5 Inhibitors: Unveiling Present and Future Perspectives. Pharmaceuticals.

[39] Bollen E., Puzzo D., Rutten K., Privitera L., De Vry J., Vanmierlo T., Kenis G., Palmeri A., D’HOoge R., Balschun D. (2014). Improved Long-Term Memory via Enhancing cGMP-PKG Signaling Requires cAMP-PKA Signaling. Neuropsychopharmacology.

[40] Rutten K., Basile J.L., Prickaerts J., Blokland A., Vivian J.A. (2008). Selective PDE inhibitors rolipram and sildenafil improve object retrieval performance in adult cynomolgus macaques. Psychopharmacology.

[41] Baratti C.M., Boccia M.M. (1999). Effects of sildenafil on long-term retention of an inhibitory avoidance response in mice. Behav. Pharmacol..

[42] Boccia M.M., Blake M.G., Krawczyk M.C., Baratti C.M. (2011). Sildenafil, a selective phosphodiesterase type 5 inhibitor, enhances memory reconsolidation of an inhibitory avoidance task in mice. Behav. Brain Res..

[43] Cuadrado-Tejedor M., Hervias I., Ricobaraza A., Puerta E., Pérez-Roldán J., García-Barroso C., Franco R., Aguirre N., García-Osta A. (2011). Sildenafil restores cognitive function without affecting β-amyloid burden in a mouse model of Alzheimer’s disease. Br. J. Pharmacol..

[44] Zhang J., Guo J., Zhao X., Chen Z., Wang G., Liu A., Wang Q., Zhou W., Xu Y., Wang C. (2013). Phosphodiesterase-5 inhibitor sildenafil prevents neuroinflammation, lowers beta-amyloid levels and improves cognitive performance in APP/PS1 transgenic mice. Behav. Brain Res..

[45] Ricciarelli R., Fedele E. (2018). cAMP, cGMP and Amyloid β: Three Ideal Partners for Memory Formation. Trends Neurosci..

[46] Zhu L., Yang J.Y., Xue X., Dong Y.X., Liu Y., Miao F.R., Wang Y.F., Xue H., Wu C.F. (2015). A novel phosphodiesterase-5 Inhibitor: Yonkenafil modulates neurogenesis, gliosis to improve cognitive function and ameliorates amyloid burden in an APP/PS1 transgenic mice model. Mech. Ageing Dev..

[47] Andoh T., Chiueh C.C., Chock P.B. (2003). Cyclic GMP-dependent protein kinase regulates the expression of thioredoxin and thioredoxin peroxidase-1 during hormesis in response to oxidative stress-induced apoptosis. J. Biol. Chem..

[48] Puzzo D., Privitera L., Palmeri A. (2012). Hormetic effect of amyloid-beta peptide in synaptic plasticity and memory. Neurobiol. Aging.

[49] Cai Z.-X., Guo H.-S., Wang C., Wei M., Cheng C., Yang Z.-F., Chen Y.-W., Le W.-D., Li S. (2016). Double-Edged Roles of Nitric Oxide Signaling on APP Processing and Amyloid-β Production In Vitro: Preliminary Evidence from Sodium Nitroprusside. Neurotox. Res..

[50] Chalimoniuk M., Strosznajder J.B. (1998). Aging modulates nitric oxide synthesis and cGMP levels in hippocampus and cerebellum. Effects of amyloid beta peptide. Mol. Chem. Neuropathol..

[51] Baltrons M.A., Pedraza C.E., Heneka M.T., García A. (2002). Beta-amyloid peptides decrease soluble guanylyl cyclase expression in astroglial cells. Neurobiol. Dis..

[52] Baltrons M.A., Pifarré P., Ferrer I., Carot J.M., García A. (2004). Reduced expression of NO-sensitive guanylyl cyclase in reactive astrocytes of Alzheimer disease, Creutzfeldt-Jakob disease, and multiple sclerosis brains. Neurobiol. Dis..

[53] Ugarte A., Gil-Bea F., García-Barroso C., Cedazo-Minguez Á., Ramírez M.J., Franco R., García-Osta A., Oyarzabal J., Cuadrado-Tejedor M. (2015). Decreased levels of guanosine 3′, 5′-monophosphate (cGMP) in cerebrospinal fluid (CSF) are associated with cognitive decline and amyloid pathology in Alzheimer’s disease. Neuropathol. Appl. Neurobiol..

[54] García-Barroso C., Ricobaraza A., Pascual-Lucas M., Unceta N., Rico A.J., Goicolea M.A., Sallés J., Lanciego J.L., Oyarzabal J., Franco R. (2013). Tadalafil crosses the blood–brain barrier and reverses cognitive dysfunction in a mouse model of AD. Neuropharmacology.

[55] Al-Amin M.M., Hasan S.N., Alam T., Hasan A.T., Hossain I., Didar R.R., Alam M.A., Rahman M.M. (2014). Tadalafil enhances working memory, and reduces hippocampal oxidative stress in both young and aged mice. Eur. J. Pharmacol..

[56] Devan B., Sierramercadojr D., Jimenez M., Bowker J., Duffy K., Spangler E., Ingram D. (2004). Phosphodiesterase inhibition by sildenafil citrate attenuates the learning impairment induced by blockade of cholinergic muscarinic receptors in rats. Pharmacol. Biochem. Behav..

[57] Orejana L., Barros-Miñones L., Jordán J., Puerta E., Aguirre N. (2012). Sildenafil ameliorates cognitive deficits and tau pathology in a senescence-accelerated mouse model. Neurobiol. Aging.

[58] Sikandaner H.E., Park S.Y., Kim M.J., Park S.N., Yang D.W. (2017). Neuroprotective effects of sildenafil against oxidative stress and memory dysfunction in mice exposed to noise stress. Behav. Brain Res..

[59] Liu S., Li X., Gao J., Liu Y., Shi J., Gong Q. (2018). Icariside II, a Phosphodiesterase-5 Inhibitor, Attenuates Beta-Amyloid-Induced Cognitive Deficits via BDNF/TrkB/CREB Signaling. Cell. Physiol. Biochem..

[60] El-Desouky S., Abdel-Halim M., Fathalla R.K., Abadi A.H., Piazza G.A., Salama M., El-Khodery S.A., Youssef M.A., Elfarrash S. (2025). A novel phosphodiesterase 5 inhibitor, RF26, improves memory impairment and ameliorates tau aggregation and neuroinflammation in the P301S tauopathy mouse model of Alzheimer’s disease. Exp. Neurol..

[61] Kang B.W., Kim F., Cho J.Y., Kim S., Rhee J., Choung J.J. (2022). Phosphodiesterase 5 inhibitor mirodenafil ameliorates Alzheimer-like pathology and symptoms by multimodal actions. Alzheimer’s Res. Ther..

[62] Park Y., Moon S., Jung H., Park S., Kim J.W., Song D.-G., In Y.-H., Han S.W., Sohn J.-H., Lee C.H. (2025). Mirodenafil improves cognitive function by reducing microglial activation and blood-brain barrier permeability in ApoE4 KI mice. Front. Aging Neurosci..

[63] Zuccarello E., Zhang H., Acquarone E., Pham D., Staniszewski A., Deng S.-X., Landry D.W., Arancio O., Fiorito J. (2023). Optimizing metabolic stability of phosphodiesterase 5 inhibitors: Discovery of a potent N-(pyridin-3-ylmethyl)quinoline derivative targeting synaptic plasticity. Bioorg. Med. Chem. Lett..

[64] Fiorito J., Vendome J., Saeed F., Staniszewski A., Zhang H., Yan S., Deng S.-X., Arancio O., Landry D.W. (2017). Identification of a Novel 1,2,3,4-Tetrahydrobenzo[b][1,6]naphthyridine Analogue as a Potent Phosphodiesterase 5 Inhibitor with Improved Aqueous Solubility for the Treatment of Alzheimer’s Disease. J. Med. Chem..

[65] Rabal O., Sánchez-Arias J.A., Cuadrado-Tejedor M., de Miguel I., Pérez-González M., García-Barroso C., Ugarte A., de Mendoza A.E.-H., Sáez E., Espelosin M. (2019). Discovery of in Vivo Chemical Probes for Treating Alzheimer’s Disease: Dual Phosphodiesterase 5 (PDE5) and Class I Histone Deacetylase Selective Inhibitors. ACS Chem. Neurosci..

[66] Cuadrado-Tejedor M., Garcia-Barroso C., Sánchez-Arias J.A., Rabal O., Pérez-González M., Mederos S., Ugarte A., Franco R., Segura V., Perea G. (2017). A First-in-Class Small-Molecule that Acts as a Dual Inhibitor of HDAC and PDE5 and that Rescues Hippocampal Synaptic Impairment in Alzheimer’s Disease Mice. Neuropsychopharmacology.

[67] Lee D.-H., Lee J.Y., Hong D.-Y., Lee E.C., Park S.-W., Na Jo Y., Park Y.J., Cho J.Y., Cho Y.J., Chae S.H. (2022). ROCK and PDE-5 Inhibitors for the Treatment of Dementia: Literature Review and Meta-Analysis. Biomedicines.

[68] Liu L., Xu H., Ding S., Wang D., Song G., Huang X. (2019). Phosphodiesterase 5 inhibitors as novel agents for the treatment of Alzheimer’s disease. Brain Res. Bull..

[69] Hainsworth A.H., Arancio O., Elahi F.M., Isaacs J.D., Cheng F. (2023). PDE5 inhibitor drugs for use in dementia. Alzheimer’s Dementia: Transl. Res. Clin. Interv..

[70] Samidurai A., Xi L., Das A., Kukreja R.C. (2023). Beyond Erectile Dysfunction: cGMP-Specific Phosphodiesterase 5 Inhibitors for Other Clinical Disorders. Annu. Rev. Pharmacol. Toxicol..

[71] Schultheiss D., Müller S.V., Nager W., Stief C.G., Schlote N., Jonas U., Asvestis C., Johannes S., Münte T.F. (2001). Central effects of sildenafil (Viagra) on auditory selective attention and verbal recognition memory in humans: A study with event-related brain potentials. World J. Urol..

[72] Grass H., Klotz T., Fathian-Sabet B., Berghaus G., Engelmann U., Käferstein H. (2001). Sildenafil (Viagra): Is there an influence on psychological performance?. Int. Urol. Nephrol..

[73] Sheng M., Lu H., Liu P., Li Y., Ravi H., Peng S.-L., Diaz-Arrastia R., Devous M.D., Womack K.B. (2017). Sildenafil Improves Vascular and Metabolic Function in Patients with Alzheimer’s Disease. J. Alzheimer’s Dis..

[74] Samudra N., Motes M., Lu H., Sheng M., Diaz-Arrastia R., Devous M., Hart J., Womack K.B. (2019). A Pilot Study of Changes in Medial Temporal Lobe Fractional Amplitude of Low Frequency Fluctuations after Sildenafil Administration in Patients with Alzheimer’s Disease. J. Alzheimer’s Dis..

[75] Goff D.C., Cather C., Freudenreich O., Henderson D.C., Evins A.E., Culhane M.A., Walsh J.P. (2009). A placebo-controlled study of sildenafil effects on cognition in schizophrenia. Psychopharmacology.

[76] Reneerkens O.A.H., Sambeth A., Van Duinen M.A., Blokland A., Steinbusch H.W.M., Prickaerts J. (2013). The PDE5 inhibitor vardenafil does not affect auditory sensory gating in rats and humans. Psychopharmacology.

[77] Reneerkens O., Sambeth A., Ramaekers J., Steinbusch H., Blokland A., Prickaerts J. (2013). The effects of the phosphodiesterase type 5 inhibitor vardenafil on cognitive performance in healthy adults: A behavioral-electroencephalography study. J. Psychopharmacol..

[78] Shim Y.S., Pae C.U., Cho K.J., Kim S.W., Kim J.C., Koh J.S. (2014). Effects of daily low-dose treatment with phosphodiesterase type 5 inhibitor on cognition, depression, somatization and erectile function in patients with erectile dysfunction: A double-blind, placebo-controlled study. Int. J. Impot. Res..

[79] Shim Y.S., Pae C.U., Kim S.W., Kim H.W., Kim J.C., Koh J.S. (2011). Effects of repeated dosing with Udenafil (Zydena) on cognition, somatization and erection in patients with erectile dysfunction: A pilot study. Int. J. Impot. Res..

[80] Choi J.B., Cho K.J., Kim J.C., Kim C.H., Chung Y.-A., Jeong H.S., Shim Y.S., Koh J.S. (2019). The Effect of Daily Low Dose Tadalafil on Cerebral Perfusion and Cognition in Patients with Erectile Dysfunction and Mild Cognitive Impairment. Clin. Psychopharmacol. Neurosci..

[81] Urios A., Ordoño F., García-García R., Mangas-Losada A., Leone P., Jose Gallego J., Cabrera-Pastor A., Megias J., Fermin Ordono J., Felipo V. (2019). Tadalafil Treatment Improves Inflammation, Cognitive Function, And Mismatch Negativity of Patients with Low Urinary Tract Symptoms and Erectile Dysfunction. Sci. Rep..

[82] Fang J., Zhang P., Zhou Y., Chiang C.-W., Tan J., Hou Y., Stauffer S., Li L., Pieper A.A., Cummings J. (2021). Endophenotype-based in silico network medicine discovery combined with insurance record data mining identifies sildenafil as a candidate drug for Alzheimer’s disease. Nat. Aging.

[83] Adesuyan M., Jani Y.H., Alsugeir D., Howard R., Ju C., Wei L., Brauer R. (2024). Phosphodiesterase Type 5 Inhibitors in Men with Erectile Dysfunction and the Risk of Alzheimer Disease: A Cohort Study. Neurology.

[84] Desai R.J., Mahesri M., Lee S.B., Varma V.R., Loeffler T., Schilcher I., Gerhard T., Segal J.B., Ritchey M.E., Horton D.B. (2022). No association between initiation of phosphodiesterase-5 inhibitors and risk of incident Alzheimer’s disease and related dementia: Results from the Drug Repurposing for Effective Alzheimer’s Medicines study. Brain Commun..

[85] Henry D.S., Pellegrino R.G. (2023). A case-control study of phosphodiesterase-5 inhibitor use and Alzheimer’s disease and related dementias among male and female patients aged 65 years and older supporting the need for a phase III clinical trial. PLoS ONE.

[86] Padma-Nathan H., Giuliano F. (2001). Oral drug therapy for erectile dysfunction. Urol. Clin. N. Am..

[87] Holt A., Blanche P., Jensen A.K., Nouhravesh N., Rajan D., Jensen M.H., El-Sheikh M., Schjerning A.M., Schou M., Gislason G. (2022). Adverse Events Associated with Coprescription of Phosphodiesterase Type 5 Inhibitors and Oral Organic Nitrates in Male Patients with Ischemic Heart Disease: A Case-Crossover Study. Ann. Intern. Med..

[88] Kloner R.A., Kostis J.B., McGraw T.P., Qiu C., Gupta A. (2022). Analysis of integrated clinical safety data of tadalafil in patients receiving concomitant antihypertensive medications. J. Clin. Hypertens..

[89] Nehra A., Jackson G., Miner M., Billups K.L., Burnett A.L., Buvat J., Carson C.C., Cunningham G.R., Ganz P., Goldstein I. (2012). The Princeton III Consensus Recommendations for the Management of Erectile Dysfunction and Cardiovascular Disease. Mayo Clin. Proc..

[90] Ausó E., Gómez-Vicente V., Esquiva G. (2021). Visual Side Effects Linked to Sildenafil Consumption: An Update. Biomedicines.

[91] Birch D.G., Toler S.M., Swanson W.H., Fish G.E., Laties A.M. (2002). A double-blind placebo-controlled evaluation of the acute effects of sildenafil citrate (Viagra) on visual function in subjects with early-stage age-related macular degeneration. Am. J. Ophthalmol..

[92] Nathoo N.A., Etminan M., Mikelberg F.S. (2015). Association Between Phosphodiesterase-5 Inhibitors and Nonarteritic Anterior Ischemic Optic Neuropathy. J. Neuroophthalmol..

[93] Zhang X.-H., Morelli A., Luconi M., Vignozzi L., Filippi S., Marini M., Vannelli G.B., Mancina R., Forti G., Maggi M. (2005). Testosterone Regulates PDE5 Expression and in vivo Responsiveness to Tadalafil in Rat Corpus Cavernosum. Eur. Urol..

[94] Sasaki H., Nagayama T., Blanton R.M., Seo K., Zhang M., Zhu G., Lee D.I., Bedja D., Hsu S., Tsukamoto O. (2014). PDE5 inhibitor efficacy is estrogen dependent in female heart disease. J. Clin. Investig..

